# AI-Driven Enhancement of Skin Cancer Diagnosis: A Two-Stage Voting Ensemble Approach Using Dermoscopic Data

**DOI:** 10.3390/cancers17010137

**Published:** 2025-01-03

**Authors:** Tsu-Man Chiu, Yun-Chang Li, I-Chun Chi, Ming-Hseng Tseng

**Affiliations:** 1 School of Medicine, Chung Shan Medical University, Taichung 402, Taiwan; cshy2085@csh.org.tw; 2 Department of Dermatology, Chung Shan Medical University Hospital, Taichung 402, Taiwan; leeyc4372@gmail.com; 3 Department of Biomedical Sciences and Engineering, National Central University, Taoyuan 320, Taiwan; jenny901918@gmail.com; 4 Information Technology Office, Chung Shan Medical University Hospital, Taichung 402, Taiwan

**Keywords:** malignant melanoma, dermoscopic images, voting ensemble learning, two-stage classification strategy

## Abstract

Background: Skin cancer is the most common cancer worldwide, with melanoma being the deadliest type, though it accounts for less than 5% of cases. Traditional skin cancer detection methods are effective but are often costly and time-consuming. Recent advances in artificial intelligence have improved skin cancer diagnosis by helping dermatologists identify suspicious lesions. Methods: The study used datasets from two ethnic groups, sourced from the ISIC platform and CSMU Hospital, to develop an AI diagnostic model. Eight pre-trained models, including convolutional neural networks and vision transformers, were fine-tuned. The three best-performing models were combined into an ensemble model, which underwent multiple random experiments to ensure stability. To improve diagnostic accuracy and reduce false negatives, a two-stage classification strategy was employed: a three-class model for initial classification, followed by a binary model for secondary prediction of benign cases. Results: In the ISIC dataset, the false negative rate for malignant lesions was significantly reduced, and the number of malignant cases misclassified as benign dropped from 124 to 45. In the CSMUH dataset, false negatives for malignant cases were completely eliminated, reducing the number of misclassified malignant cases to zero, resulting in a notable improvement in diagnostic precision and a reduction in the false negative rate. Conclusions: Through the proposed method, the study demonstrated clear success in both datasets. First, a three-class AI model can assist doctors in distinguishing between melanoma patients who require urgent treatment, non-melanoma skin cancer patients who can be treated later, and benign cases that do not require intervention. Subsequently, a two-stage classification strategy effectively reduces false negatives in malignant lesions. These findings highlight the potential of AI technology in skin cancer diagnosis, particularly in resource-limited medical settings, where it could become a valuable clinical tool to improve diagnostic accuracy, reduce skin cancer mortality, and reduce healthcare costs.

## Simple Summary

This study utilized datasets from two ethnic groups to develop an AI diagnostic model. This model was trained using transfer learning, leveraging eight pre-trained models, including convolutional neural networks and vision transformers. The three-class AI model assists doctors in distinguishing between patients with melanoma who require urgent treatment, those with non-melanoma skin cancers who can be treated later, and benign cases that do not require intervention. The proposed two-stage classification strategy significantly improved diagnostic accuracy and reduced false negatives. This research demonstrates the success of the proposed method in both datasets. These findings highlight the potential of AI technology in skin cancer diagnosis, particularly in resource-limited medical settings, where it could become a valuable clinical tool to improve diagnostic accuracy, reduce skin cancer mortality, and decrease healthcare costs.

## 1. Introduction

Skin cancer is widespread around the world and is one of the most common cancers in humans. On the basis of histopathological classification, it can mainly be divided into melanoma and non-melanoma skin cancers. Although melanoma has a lower incidence, it is the most lethal type of skin cancer. Non-melanoma skin cancers, which include basal cell carcinoma and squamous cell carcinoma (collectively known as keratinocyte carcinomas), have a higher incidence but relatively lower mortality rates. Melanoma is the deadliest form of skin cancer [1,2]. Due to the highly malignant nature of melanoma, early diagnosis is crucial to improve treatment outcomes. The prognosis and burden of skin cancer are highly dependent on the type of cancer and the stage at which it is diagnosed. Early diagnosis can greatly increase the chances of successful treatment and potentially reduce the need for surgery, radiation therapy, chemotherapy, targeted therapy, or immunotherapy, thus improving patient care and reducing healthcare care costs. The dermoscopic examination has become an essential tool in diagnosing melanoma and other pigmented skin lesions, with computer-aided skin lesion classification methods based on dermoscopic images receiving significant attention [3], especially in distinguishing melanoma from other benign skin lesions. However, due to the high visual similarity between different skin lesions, relying solely on visual observation and experience for diagnosis presents certain challenges and limitations. Beyond dermoscopic imaging, novel approaches, such as hyperspectral imaging (HSI), are gaining traction in skin cancer diagnostics. Lin et al. [4] proposed the spectrum-aided visual enhancer (SAVE), which converts RGB images into hyperspectral images and has demonstrated remarkable performance using the YOLO framework. With the advancement of artificial intelligence technologies, computer-aided diagnostic systems based on dermoscopic images have emerged as a potential solution. This study proposes an ensemble model, refined through various attempts, to improve the accuracy of predicting benign and malignant skin lesions, particularly melanoma.

To assess the effectiveness of AI models in dermoscopy images, numerous researchers rely on public datasets such as PH2, MED-NODE, and ISIC. This research examined 30 recent studies focused on computer-aided diagnosis (CAD) of melanoma for binary classification tasks, published from 2016 to 2024, and provided the minimum and maximum values for four performance evaluation metrics, as illustrated in Table 1.

The aim of this study is to enhance the accuracy of diagnosing malignant melanoma and other skin lesions from dermoscopic images by leveraging multiple pre-trained deep-learning models combined with an ensemble model strategy. Specifically, using a two-stage classification approach, this study seeks to reduce the false negative rate in the diagnostic process, which is of significant clinical importance. By conducting precise model training and validation, this research aims to provide clinicians with an effective auxiliary diagnostic tool that allows accurate identification of lesions that require immediate attention at an early stage.

## 2. Materials

### 2.1. CSMUH Dataset

This study used the CSMUH dataset, which was compiled by dermatologists at Chung Shan Medical University Hospital and comprises dermoscopic images of skin lesions collected from 2019 to 2023. Photos were obtained from individuals who had skin lesions excised in the dermatology department, with all lesions verified by pathologists at the Chung Shan Medical University Hospital. The inclusion criteria encompassed patients aged 18 years or older who consented to clinical imaging of skin lesions without regard to gender while excluding children and special groups. Exclusion criteria included photos that did not meet diagnostic classification, were unclear or indeterminate, or had dubious pathological diagnoses. This study used a Canon EOS 500D camera and a HEINE DELTA 20 dermatoscope. Photographs may or may not include the built-in scale of the dermatoscope. The lesions were cropped to the center of the image and must be clearly visible. Natural exposure settings were used, with no post-processing adjustments made to aspects such as color tone, sharpness, or saturation. Throughout the data collection phase, the dataset was classified into seven categories according to the ISIC framework, with subsequent categorization and case counts. There were 111 cases of melanoma, 199 cases of melanocytic nevus, 45 cases of basal cell carcinoma, 82 cases of actinic keratosis (and Bowen’s disease, keratoacanthoma, and squamous cell carcinoma), 134 cases of benign keratosis, 42 cases of dermatofibroma and 53 cases of vascular lesions, which is a total of 666 cases.

According to the objectives of this study, our objective was to identify key malignant melanomas by reclassifying the seven categories in the ISIC database into three distinct groups as follows: melanoma was classified as category 2, basal cell carcinoma and actinic keratosis (and Bowen’s disease, keratoacanthoma, and squamous cell carcinoma) were combined into category 1, and all other categories were grouped into category 0. In this classification system, a higher category number indicates a more severe condition. The machine learning model was then trained using this three-category classification. The number of instances in each category after reclassification is presented in Table 2.

Furthermore, to improve accuracy and reduce false negatives, we designed a two-stage voting process. In this approach, instances classified as category 0 by the first AI model were further screened by a second AI model, which was trained to reclassify the seven ISIC categories into two groups: required treatment (malignant and premalignant) and no treatment required (benign). Possible treatment-required cases were classified as category 1, and benign cases as category 0. The number of cases in each category after this reclassification is shown in Table 3.

### 2.2. ISIC Dataset

This study performed data integration based on the ISIC2018 Task 3 dataset [28]. Due to the problem of data imbalance in this dataset, additional data from ISIC2017 [29], ISIC2018 Task 1 [30], ISIC2019 [28,31], and ISIC2020 [32] to include the less represented skin disease categories. After removing duplicate images, these datasets were combined into a comprehensive ISIC 2017–2020 dataset for further research.

The number of instances for each category in the ISIC 2017–2020 dataset is as follows: 5008 cases of melanoma, 6705 cases of melanocytic nevus, 3261 cases of basal cell carcinoma, 1043 cases of actinic keratosis, 2657 cases of benign keratosis, 236 cases of dermatofibroma, and 253 cases of vascular lesions, totaling 19,163 cases. These categories were reclassified as follows: melanoma was classified as category 2, basal cell carcinoma and actinic keratosis were combined into category 1, and all other categories were combined into category 0. In this classification, a higher category number indicates a more severe condition. The number of cases in each category after reclassification is shown in Table 4. Furthermore, the categories were divided into benign and malignant groups, with malignant cases classified as category 1 and benign cases as category 0. The number of cases in each category after this reclassification is shown in Table 5.

## 3. Methods

### 3.1. Research Structure

**Figure 1:**
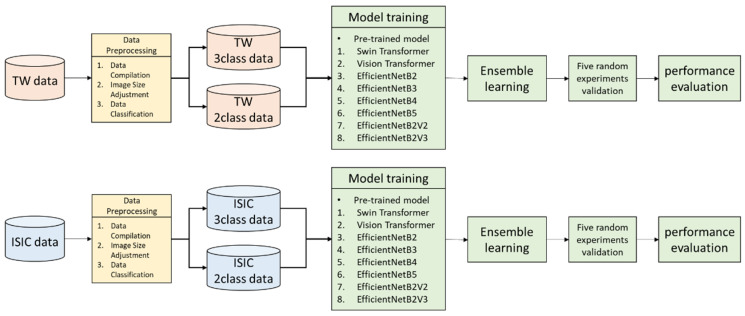
Research flow chart.

In this study, the research flow chart is shown in Figure 1. Both the ISIC dataset and the CSMUH dataset were used for model training and testing, with 19,163 and 666 samples, respectively. The dermoscopic images were first preprocessed by resizing all images to 224 × 224 pixels and normalizing them to facilitate subsequent deep-learning model computations. The training set was augmented by randomly flipping, rotating, translating, and scaling the dermoscopic images. This increases the amount of data from a small number of categories and brings the training set closer to a balanced distribution of categories. After completion of image preprocessing, transfer learning was applied to integrate an ensemble model for training. The model was validated using five random experiments, with the primary evaluation metric being accuracy (ACC), calculated as shown in Equation (1). Here, TP represents true positives where the model correctly identifies positive samples, FN represents false negatives where the model incorrectly predicts positive samples as negative, FP represents false positives where the model incorrectly predicts negative samples as positive, and TN represents true negatives where the model correctly identifies negative samples.

ACC = (TP + TN)/(TP + TN + FP + FN)(1)

To further improve the classification performance of the AI model, this study conducted separate model training for categories of three classes and two classes on the same dataset. Subsequently, images predicted as category 0 in the three-class test set were re-evaluated using the two-class model. This two-stage classification strategy effectively reduces the false-negative rate of the model.

### 3.2. Data Preprocessing

The dataset was divided into the training set and the test set, with all images resized to 224 × 224 pixels and normalized to facilitate computations in the deep learning model. To enhance the volume of image data during preprocessing, data augmentation techniques [33], such as random flipping, rotation, translation, and scaling, were applied to the training dataset.

### 3.3. Transfer Learning

Transfer learning involves applying a model originally trained in one domain to a new domain, transferring its learned knowledge to the target task without the need to train the model from scratch. The advantage of this approach is the reduced requirement for large amounts of training data. In this study, fine-tuning was performed on pre-trained models by fixing the weights of specific layers in the earlier parts of the model while training the later layers to obtain new weight values. Eight different pre-trained models were selected for this study, including EfficientNetB2, EfficientNetB3, EfficientNetB4, EfficientNetB5 [34], EfficientNetV2B2, EfficientNetV2B3 [35], ViT [36], and Swin [37].

### 3.4. Ensemble Learning

This study employed ensemble learning techniques to enhance the predictive performance of models by combining different models to achieve better overall results. Specifically, the voting ensemble method [38,39] was used, where the outputs of multiple independent models were combined as inputs for a new model, which was then trained as a new model. In this technique, multiple models are trained independently, and their predictions are combined through a voting process. Each model makes a prediction for each input sample, and the final output prediction is determined by a majority vote among all models. This approach is commonly used to take advantage of the strengths of multiple learning algorithms, improve predictive performance, and reduce the likelihood of errors from any single model.

### 3.5. Model Architecture

**Figure 2:**
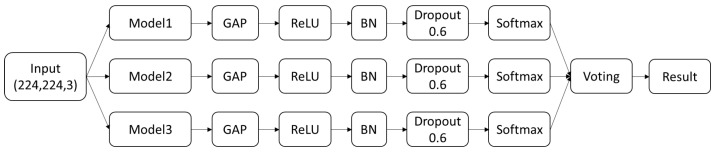
Model architecture.

In this study, an ensemble deep learning model was constructed based on three pre-trained models using the voting method. The top three pre-trained models with the best training performance were selected, and a fully connected layer with a ReLU activation function and a Global Average Pooling (GAP) layer were added to each. To avoid overfitting, Batch Normalization (BN) and Dropout layers were incorporated. After obtaining the prediction results for the skin lesions of each model, a voting process was applied to generate the final prediction result. The model architecture is illustrated in Figure 2.

## 4. Results

### 4.1. Step 1: Three-Class Classification

#### 4.1.1. CSMUH Dataset

**Figure 3:**
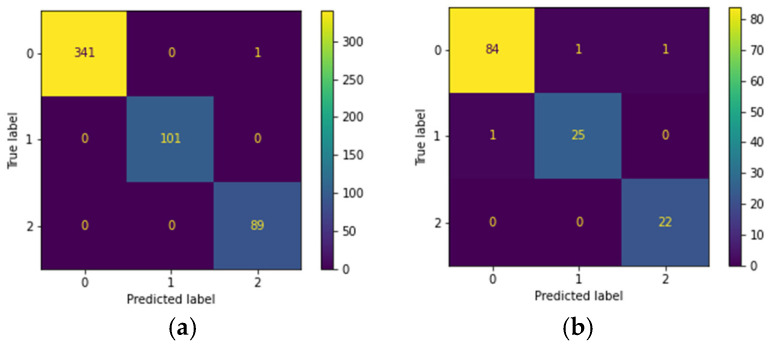
Confusion matrix of the CSMUH dataset: (**a**) three-class training set; (**b**) three-class test set.

This study explored the performance of various deep learning models in the classification of skin lesions, including the Swin Transformer, vision transformer (ViT), ResNet50, VGG16, and multiple versions of EfficientNet, and combined these models into an ensemble model. The optimal ensemble model consisted of the Swin Transformer, vision transformer (ViT), and EfficientNetB5, with its best performance displayed in the confusion matrix shown in Figure 3.

As shown in Table 6, the ensemble model achieved an accuracy of 99.77% during the training phase and 97.31% during the testing phase. This outperformed individual models, such as the Swin Transformer, which had a training accuracy of 98.46% and a testing accuracy of 89.85%; the vision transformer (ViT) model, which reached 99.47% accuracy during training and 90% during testing; the ResNet50 model, which had a training accuracy of 98.72% and a testing accuracy of 86.12%; the VGG16 model, which reached 75.71% accuracy during training and 68.36% during testing; and the EfficientNetB5 model, which achieved 99.36% accuracy in training and 89.7% in testing. The ensemble model demonstrated superior performance compared to these single models.

#### 4.1.2. ISIC Dataset

**Figure 4:**
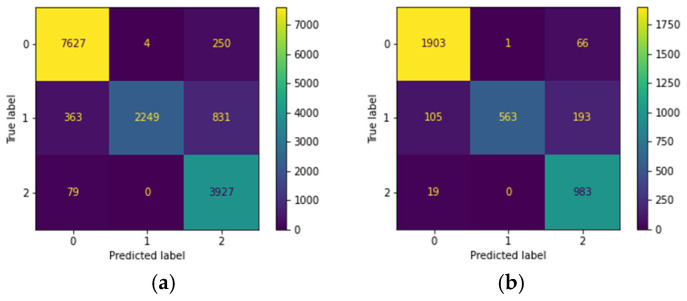
Confusion matrix of the ISIC dataset: (**a**) three-class training set; (**b**) three-class test set.

In this study, the performance of various deep learning architectures was evaluated in the skin lesion classification task, including the Swin Transformer, the vision transformer (ViT), and multiple versions of EfficientNet. These models were combined into an ensemble model. The optimal ensemble model consisted of the Swin Transformer, EfficientNetB5, and EfficientNetV2B2, with its best performance illustrated by the confusion matrix shown in Figure 4.

As shown in Table 7, the ensemble model achieved an accuracy of 95.86% during the training phase and 85.38% during the testing phase. This outperformed individual models, such as the Swin Transformer, which had a training accuracy of 87.05% and a testing accuracy of 77.43%; the EfficientNetV2B2 model, which achieved 90.62% accuracy during training and 81.27% during testing; and the EfficientNetB5 model, which achieved 95.78% accuracy during training and 85.09% during testing. The ensemble model demonstrated superior performance compared to these single models.

### 4.2. Step 2: Two-Stage Strategy

#### 4.2.1. CSMUH Dataset

**Figure 5:**
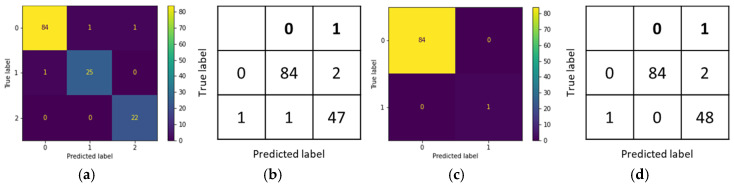
Confusion matrix of the CSMUH test set: (**a**) the three-class model; (**b**) the three-class model converted to binary classification; (**c**) binary classification after identifying benign cases in the three-class model; (**d**) binary classification of the two-stage model.

This study used a two-stage model classification strategy to reduce the false negative rate and improve the diagnostic accuracy of skin lesions. As shown in Figure 5, the initial three-class classification results (Figure 5a) correctly identified 84 benign labels as benign; however, one malignant label was incorrectly classified as benign. In the second stage of testing, all cases initially classified as benign were re-evaluated using a binary classification to distinguish between benign and malignant. The results of this stage (Figure 5c) further confirmed the accuracy of the 84 benign cases and successfully corrected the previous misclassification, reducing the number of false negatives to zero. This validation method effectively minimized the occurrence of false negatives, not only in diagnostic accuracy but also in the applicability and reliability of the model in clinical practice.

#### 4.2.2. ISIC Dataset

**Figure 6:**
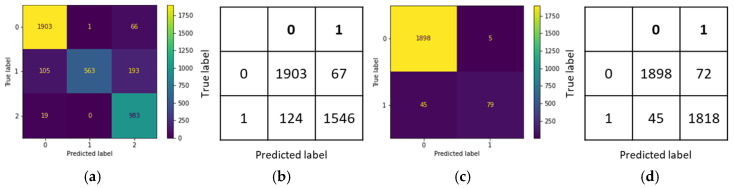
Confusion matrix of the ISIC test set: (**a**) the three-class model; (**b**) the three-class model converted to binary classification; (**c**) binary classification after identifying benign cases in the three-class model; (**d**) binary classification of the two-stage model.

In this study, a two-stage model classification approach was employed to reduce the false negative rate and thus improve the accuracy of skin lesion diagnosis. As shown in Figure 6, the confusion matrix on the left shows the results of the three-class classification, where 1903 benign labels were correctly predicted as benign, but 124 malignant cases were misclassified as benign. In the second stage of testing, all images initially classified as benign were re-evaluated using a binary classification (benign versus malignant). The confusion matrix on the right shows that at this stage, 1898 benign labels were correctly identified, and the number of originally misclassified malignant cases was reduced from 124 to 45. This demonstrates that further validation can reduce the occurrence of false negatives. This method not only improved diagnostic accuracy but also enhanced the reliability of the model’s application in clinical practice. 

#### 4.2.3. Comparison of Performance Improvement Using the Two-Stage Strategy

To further illustrate the effectiveness of the two-stage classification strategy proposed in this study, Table 8 presents the values of five binary classification evaluation metrics: accuracy, Sensitivity, Specificity, false negative rate, and false positive rate. These metrics are evaluated for three AI classification models: the individual three-class model, the ensemble three-class model, and the two-stage model, all based on the CSMUH test set. Table 9 displays the performance comparison results for the ISIC test set. Whether considering the CSMUH dataset for the Eastern population or the ISIC dataset for the Western population, the experimental results in Table 8 and Table 9 clearly indicate that the two-stage model exhibits the best classification performance. It is followed by the ensemble three-class model in second place and the individual three-class model, which demonstrates the worst classification performance. Using the critical false negative rate (FNR) indicator in medical diagnosis as an example, in the CSMUH test set, the FNR indicator is 0.128 in the individual three-class model. In the ensemble three-class model, it is 0.021, and in the two-stage model, it drops to 0. In the ISIC test set, the FNR indicator is 0.151 in the individual three-class model, 0.021 in the ensemble three-class model, and drops to 0.024 in the two-stage model. It is worth noting that Table 6 and Table 7 show the average accuracy of five experiments, while Table 8 and Table 9 display the best accuracy from one of the five experiments.

## 5. Discussion

This study demonstrates the potential of ensemble models in dermoscopic images, particularly in significantly reducing the false negative rate in the diagnosis of malignant melanoma. The study utilized two datasets and trained eight different pre-trained model architectures, selecting the top three models to fine-tune and create a highly stable and reliable ensemble model. Compared to traditional single-model approaches, the ensemble model combines the strengths of multiple models, thus improving diagnostic accuracy and stability.

The study used a two-stage model classification strategy: First, a three-class classification was performed to identify benign cases, and then these benign cases were subtracted into a second-stage binary classification to differentiate between benign and malignant. In the CSMUH dataset, this two-stage classification strategy reduced the number of misclassified malignant cases to zero; in the ISIC dataset, it significantly reduced the number of malignant cases misclassified as benign from 124 to 45. Although some malignant lesions were initially misclassified as benign in the three-class classification, subsequent binary verification significantly reduced this number, demonstrating good performance. This approach not only improved diagnostic accuracy but also improved the model’s applicability and reliability in clinical practice.

Through this study, a highly stable and reliable ensemble model was established. Using a two-stage classification method, starting with a three-class classification followed by binary verification, the model performance was significantly improved. This approach achieved the best accuracy in predicting malignant melanoma from dermoscopic images and effectively reduced the false negative rate of malignant skin tumors. Clinically, this could provide substantial benefits compared to traditional diagnostic methods. The AI model developed in this study not only improved diagnostic accuracy but also significantly reduced the risk of misdiagnosis, particularly in controlling false negatives, which is crucial in clinical applications, as false negatives could lead to delayed treatment with severe consequences for patients. This study offers clinicians a tool to help identify lesions that require further examination and treatment at an early stage (malignant melanoma or malignant skin lesions), potentially reducing mortality rates and healthcare costs.

This research presents an effective method to accurately predict benign and malignant skin lesions, helping patients and dermatologists identify skin lesions early, thus reducing misdiagnosis rates and improving treatment efficiency.

However, there are some limitations to this study. For example, although the ensemble model showed good diagnostic performance, its computational demands are relatively high, which may limit its application in resource-constrained medical environments. Additionally, the CSMUH dataset used in this study is relatively small, which may affect the model’s generalizability across different populations. Future research should focus on expanding the dataset and incorporating more data from diverse sources to validate the model’s applicability across broader populations.

Table 10 provides a comparative summary of recent techniques for the binary classification of melanoma from 2021 to the present. It includes information on the authors, year, method, validation, dataset, classification, and overall accuracy of the test set. Establishing valid comparisons is challenging due to the use of different datasets, varying sizes, and various performance metrics across various studies. However, the two-stage voting ensemble approach proposed in this study demonstrates outstanding performance.

In general, this study suggests that the use of AI technology to aid in the diagnosis of skin lesions is a promising direction, particularly in the early diagnosis of melanoma and in the identification of benign and malignant skin lesions. The application of ensemble models helps improve diagnostic precision and reduce misdiagnosis rates, thus improving patient outcomes.

## 6. Conclusions

This study effectively improved the accuracy of diagnosing benign and malignant skin lesions in dermoscopic images using ensemble learning and a two-stage classification strategy, particularly achieving significant success in predicting malignant melanoma. First, the results demonstrated that the three-class AI model can assist doctors in distinguishing between melanoma patients who require urgent treatment, non-melanoma skin cancer patients who can be treated later, and benign cases that do not require intervention. Subsequently, the two-stage classification strategy effectively reduced the false negative rate during the diagnostic process. In particular, in the CSMUH dataset, the model achieved 100% accuracy in detecting malignant lesions, completely eliminating false negatives. This outcome not only underscores the potential of AI technology in the diagnosis of skin lesions but also provides clinicians with a reliable auxiliary diagnostic tool to help them more accurately identify lesions that require immediate attention at an early stage. This could potentially reduce skin cancer-related mortality rates and save healthcare costs.

Future research should focus on expanding the dataset and further optimizing the model, particularly improving its computational efficiency and adaptability in resource-limited environments. In general, this study provides strong evidence for the clinical value of AI-assisted skin lesion diagnosis, with significant potential for practical application.

## 7. Future Work

Future research should focus on improving the extraction of features and the annotation of dermoscopic images to further improve the diagnostic accuracy of AI models. Furthermore, the incorporation of clinical data such as age, sex, and location of the lesion into the models could improve diagnostic performance, particularly in primary care settings, where it would improve the precision of early diagnoses and reduce unnecessary procedures. Expanding the size of the dataset to include more pathologically confirmed skin lesion images would also help improve the generalizability of the model. Future studies should explore the cost-effectiveness of AI technology in the early detection of skin cancer and its acceptance in clinical practice, as these will be key factors in determining the widespread adoption of AI technology.

## Figures and Tables

**Table 1:** Results of the performance evaluation test on the models’ melanoma binary classification of melanoma.

Authors	Dataset	AUC	Accuracy	Sensitivity	Specificity
[5,6,7]	PH2	NA	0.861~0.975	0.790~0.981	0.925~0.938
[8]	Subset of PH2	NA	0.950	0.925	0.966
[9]	ISIC 2016	0.766	0.818	0.818	0.714
[9,10,11]	ISIC 2017	0.870~0.964	0.857~0.933	0.490~0.933	0.872~0.961
[10,12,13,14,15,16,17,18,19]	ISIC 2018	0.847~0.989	0.803~0.938	0.484~0.888	0.957~0.978
[20,21]	Subset of ISIC 2018	0.970	0.880~0.910	0.920~0.960	NA
[10,15]	ISIC 2019	0.919~0.991	0.896~0.924	0.483~0.896	0.976~0.977
[8,22]	Subset of ISIC 2019	0.942	0.870~0.930	0.920~0.925	0.820~0.933
[13,14,23,24]	Combined	0.880~0.960	0.803~0.950	0.851~0.930	0.844~0.950
[25]	Subset of combining ISIC 2018 and ISIC 2019	0.981	0.965	0.878	0.993
[26]	MED-NODE	0.810	NA	0.810	0.800
[27]	Subset of ISBI 2017	0.891	0.866	0.556	0.785

**Table 2:** The number of cases in each category for the three-class classification of CSMUH.

Class	Num	Label	Image
Melanoma (mel)	111	2	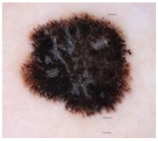
Basal cell carcinoma (bcc) and actinic keratosis (ak) (and Bowen’s disease, keratoacanthoma, and squamous cell carcinoma)	127	1	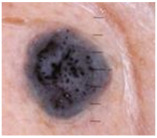
Melanocytic Nevus (nv), benign keratosis (bkl), dermatofibroma (df), and vascular (vasc)	428	0	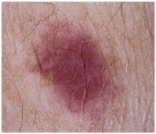

**Table 3:** The number of cases in each category for the two-class classification of CSMUH.

Class	Num	Label	Image
Melanoma (mel), basal cell carcinoma (bcc), and actinic keratosis (ak) (and Bowen’s disease, keratoacanthoma, and squamous cell carcinoma)	238	1	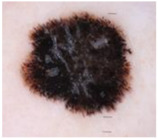
Melanocytic nevus (nv), benign keratosis (bkl), dermatofibroma (df), and vascular (vasc)	428	0	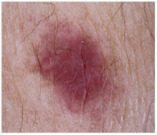

**Table 4:** The number of cases in each category for the three-class ISIC classification.

Class	Num	Label	Image
Melanoma (mel)	5008	2	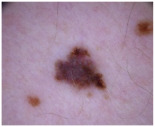
Basal cell carcinoma (bcc) and Actinic keratosis/intraepithelial carcionma (akiec)	4304	1	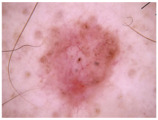
Melanocytic nevus (nv), benign keratosis (bkl), dermatofibroma (df), and vascular (vasc)	9851	0	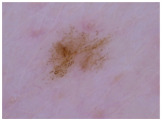

**Table 5:** The number of cases in each category for the two-class classification of ISIC.

Class	Num	Label	Image
Melanoma (mel), basal cell carcinoma (bcc), and actinic keratosis/intraepithelial carcinoma (akiec)	9312	1	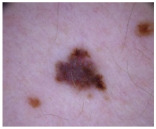
Melanocytic nevus (nv), benign keratosis (bkl), dermatofibroma (df), and vascular (vasc)	9851	0	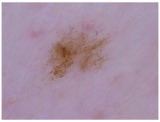

**Table 6:** Results of training and prediction of the three-class model for skin disease of CSMUH.

Model	Train ACC	Test ACC
Swin Transformer	0.9846 ± 0.0108	0.8985 ± 0.0224
Vision Transformer	0.9947 ± 0.0030	0.9000 ± 0.0304
EfficientNetB2	0.9805 ± 0.0040	0.8776 ± 0.0421
EfficientNetB3	0.9850 ± 0.0106	0.8776 ± 0.0121
EfficientNetB4	0.9914 ± 0.0068	0.8687 ± 0.0180
EfficientNetB5	0.9936 ± 0.0019	0.8970 ± 0.0256
EfficientNetV2B2	0.9820 ± 0.0089	0.8701 ± 0.0342
EfficientNetV2B3	0.9594 ± 0.0307	0.8463 ± 0.0516
ResNet50	0.9872 ± 0.0025	0.8612 ± 0.0312
VGG16	0.7571 ± 0.2763	0.6836 ± 0.2280
Ensemble	0.9977 ± 0.0022	0.9731 ± 0.0060

**Table 7:** Results of the training and test of the three-class model for ISIC skin disease.

Model	Train ACC	Test ACC
Swin Transformer	0.8705 ± 0.0392	0.7743 ± 0.0310
Vision Transformer	0.7957 ± 0.2867	0.7103 ± 0.2442
EfficientNetB2	0.8329 ± 0.1079	0.7459 ± 0.0979
EfficientNetB3	0.6368 ± 0.2338	0.5845 ± 0.2033
EfficientNetB4	0.8563 ± 0.0810	0.7703 ± 0.0708
EfficientNetB5	0.9578 ± 0.0089	0.8509 ± 0.0072
EfficientNetV2B2	0.9062 ± 0.0529	0.8127 ± 0.0426
EfficientNetV2B3	0.8333 ± 0.2850	0.7539 ± 0.2452
Ensemble	0.9586 ± 0.0079	0.8538 ± 0.0095

**Table 8:** Comparison of performance improvement using the two-stage strategy at CSMUH test set.

CSMUH Model		Accuracy (ACC)	Sensitivity	Specificity	False Negative Rate	False Positive Rate
Three-class	Individual	0.940	0.872	0.977	0.128	0.023
	Ensemble	0.978	0.979	0.977	0.021	0.023
Two-stage	Ensemble	0.985	1.000	0.977	0.000	0.023

**Table 9:** Comparison of performance improvement using the two-stage strategy at ISIC test set.

ISIC Model		Accuracy (ACC)	Sensitivity	Specificity	False Negative Rate	False Positive Rate
Three-class	Individual	0.862	0.849	0.873	0.151	0.127
	Ensemble	0.900	0.926	0.880	0.074	0.120
Two-stage	Ensemble	0.969	0.976	0.963	0.024	0.037

**Table 10:** A comparative summary of recent techniques for binary classification of melanoma.

Author, Year	Method	Validation	Dataset	Class	Test ACC
Raza, R., et al. [40], 2021	Ensemble with Xception, Inception-ResNet-V2, DenseNet121, DenseNet201	Holdout (7:1:2) full: 724	Dongsan Clinic in KeiMyung University Daegu, Korea	2	0.979
Alfi IA, R.M., Shorfuzzaman M, Nazir A. [21], 2022	Ensemble with MobileNet, Xception, ResNet50, ResNet50V2, and DenseNet121	Holdout (8:2) full: 3297	Subset of ISIC 2018	2	0.910
Chang C-C, L.Y.-Z., Wu H-C, Tseng M-H. [25], 2022	InceptionResNetV2 + XGB + K-means SMOTE	Holdout (8:2) full: 2299	Subset of combining ISIC 2018 and ISIC 2019	2	0.965
Wu H-C, T.Y.-C., Chen P-H, Tseng M-H. [18], 2023	MEL-HSNet	Holdout (9:1) full: 4331	ISIC 2018	2	0.938
Roshni Thanka, M., et al. [41], 2023	VGG16 + XGBoost	5-StratifiedKFold train: 1000 test: 416	ISIC	2	0.991
	VGG16 + LightGBM			2	0.972
Azeem, M., et al. [42], 2023	SkinLesNet VGG16 ResNet50	Holdout (8:2) full: 1314	PAD-UFES-20-Modified dataset	3	0.790
					0.820
					0.960
Qasim Gilani, S., et al. [43], 2023	Spiking VGG-13	Holdout (70:15:15) full: 6993	ISIC 2019	2	0.896
Hossain, M.M., et al. [44], 2023	Ensemble (max voting) with MobileNetV2, AlexNet, vgg16, ResNet50, DenseNet121, DenseNet201, InceptionV3, ResNet50V2, Inception, ResNetV2, Xception	Holdout train: 2597 validation: 100 test: 1000	ISIC 2018	2	0.932
Thwin, S.M. and H.-S. Park [45], 2024	Ensemble with VGG, ResNet-50, and Inception-V3.	Holdout (75:25) full: 995	ISIC	3	0.910
Faghihi, A.F., M.; Rajabi, R. [46], 2024	VGG19	10-fold Cross Validation full: 2541	ISIC	2	0.987
Our approach, 2024	Voting Ensemble with Swin, EfficientNetB5, and EfficientNetV2B2	Holdout (8:2) full:19,163	ISIC 2017~2020	3	0.900
	Two-Stage Voting Ensemble with Swin, EfficientNetB5, and EfficientNetV2B2	Holdout (8:2) full:19,163	ISIC 2017~2020	2	0.969
	Voting Ensemble with Swin, ViT, and EfficientNetB5	Holdout (8:2) full: 666	CSMUH	3	0.978
	Two-Stage Voting Ensemble with Swin, ViT, and EfficientNetB5	Holdout (8:2) full: 666	CSMUH	2	0.985

## Data Availability

The ISIC data that support the findings of this study are available in [ISIC Archive] at [https://www.isic-archive.com/ (accessed on 1 January 2024)], reference numbers [27,28,29,30,31]. The CSMUH data that support the findings of this study are available from the corresponding author upon reasonable request.
